# *Debaryomyces hansenii* Reshapes the Fungal Community of Iberian Cured Pork Loin: An ITS1 Metabarcoding Approach

**DOI:** 10.3390/microorganisms14051113

**Published:** 2026-05-14

**Authors:** Helena Chacón-Navarrete, Marina Barbudo-Lunar, Francisco Javier Ruiz-Castilla, José Ramos

**Affiliations:** 1 Department of Agricultural Chemistry, Edaphology and Microbiology, University of Córdoba, 14071 Córdoba, Spain; b62chnah@uco.es (H.C.-N.); a32rucaf@uco.es (F.J.R.-C.); 2 Department of Biochemistry and Molecular Biology, University of Córdoba, 14071 Córdoba, Spain; b62balum@uco.es

**Keywords:** *Debaryomyces hansenii*, Iberian pork loin, biocontrol, rDNA sequencing

## Abstract

Increasing consumer demand for natural and safe food products has led to the exploration of biocontrol alternatives to chemical preservatives, especially in the cured meat industry. The yeast *Debaryomyces hansenii* has emerged as a promising biocontrol candidate due to its antagonistic properties against spoilage fungi. This study assessed the impact of *D. hansenii* inoculation on the fungal community structure of Iberian cured pork loin using high-throughput sequencing of the *ITS1* region. Ion Torrent ITS1 amplicon sequencing, QIIME2/DADA2 pipeline, and ALDEx2 differential abundance analysis were applied to this study. Pork loin samples inoculated with *D. hansenii* were compared to non-inoculated controls to evaluate changes in the fungal microbiome. Inoculation resulted in a marked decrease in fungal diversity and evenness, indicating strong competition by *D. hansenii* against native fungal populations. This effect was reflected in a significant reduction in alpha diversity in inoculated samples (Shannon, *p* = 0.0042; Pielou *p* = 0.0075; Gini–Simpson, *p* = 0.0081). Notably, genera associated with spoilage and mycotoxin production, particularly *Aspergillus* and *Penicillium*, were significantly reduced in inoculated samples. Simultaneously, *D. hansenii* became dominant, reducing other yeasts and filamentous fungi. These findings highlight the powerful competitive and biocontrol potential of *D. hansenii*, demonstrating its ability to improve microbial safety by potentially reducing mycotoxin-associated risks through the suppression of toxigenic genera. This is the first study to characterise the fungal community of Iberian pork loin using metabarcoding under *D. hansenii* inoculation. The findings confirm that the inoculation of *D. hansenii* can substantially reduce fungal contamination risks. Overall, the results contribute valuable insights into microbial interactions during meat curing and underscore the practical benefits of targeted starter cultures for enhancing food safety and quality.

## 1. Introduction

Spanish Iberian pork loin, known as “lomo ibérico”, is a cherished element of Spanish gastronomy, celebrated for its distinctive flavour and texture. This cured meat product is made from the loin of Iberian pigs, which are often acorn-fed, contributing to the meat’s rich taste and marbling. The preparation involves marinating the pork loin in a mixture of salt, paprika, and garlic, then stuffing it into a natural pork casing before curing it for several months. This process imparts a delicate, slightly salty flavour with a hint of paprika, making it a popular choice in tapas and other traditional dishes [1,2].

Historically, Iberian pork loin has been produced in regions like Extremadura and Salamanca, where the climate and traditional methods favour the curing process. The unique environment and artisanal techniques used in these areas ensure the high quality and distinctive characteristics of the product. Its preparation follows traditional techniques including salting, preservative addition, and dehydration. The drying and ripening stages are critical for ensuring product safety [3]. Consumers highly value these products, particularly when they are associated with natural rearing conditions and high animal welfare standards [4]. Industrial production also exists, where cold smoking and adding nitrates and phosphates help preserve the meat and enhance its flavour and colour. Microbial communities in Iberian pork loin and other cured meat products play a crucial role in both the safety and the sensory qualities of these foods. Understanding the types of microorganisms present, their effects on the product, and proper management during processing is essential for ensuring product quality and safety. Beneficial microorganisms found in Iberian pork loin include various strains of lactic acid bacteria, bifidobacteria, and *Staphylococcus* species, as well as yeasts like *Debaryomyces hansenii* [3,5,6]. These microorganisms contribute to the probiotic potential, safety, and sensory qualities of the product, making them valuable for the production and enhancement of Iberian pork loin. Nevertheless, there are several undesired bacterial species belonging to genera such as *Streptococcus*, *Trueperella*, *Salmonella*, *Campylobacter* and *Listeria* among others, and multiple studies have explored strategies to reduce their presence [3,7].

Cured meat products are also susceptible to colonisation by various fungal species, which can have both beneficial and detrimental effects on the product. Certain fungi, such as *Penicillium chrysogenum* and *Debaryomyces hansenii*, contribute positively to the flavour and texture of dry-cured meats by producing volatile compounds and enhancing proteolysis and lipolysis [8,9]. The predominant fungal genera found in cured meats include *Penicillium*, *Aspergillus*, and *Cladosporium*. These fungi often originate from the production environment, including air, surfaces, and equipment [10]. Some species of these genera of fungi are especially worrying as they are mycotoxins producers. Mycotoxins are toxic secondary metabolites produced by certain fungi, particularly moulds, that can contaminate food and feed, posing significant health risks to humans and animals. These compounds are known for their diverse toxicological effects, including carcinogenic, nephrotoxic, hepatotoxic, immune-suppressive, and mutagenic properties [11]. Controlling unwanted fungal growth, particularly mycotoxin-producing species like *Aspergillus* and *Penicillium*, is essential for ensuring both consumer health and product safety. Thus, exploring biological alternatives for fungal suppression has become critically important.

Among fungi, yeasts also play a crucial role in the curing process as previously mentioned. *D. hansenii* is a yeast species commonly found in dry-cured meat products. It has been studied for its potential to improve the safety and quality of these products by inhibiting the growth of harmful moulds and affecting the sensory characteristics of the meat. The interactions between *D. hansenii* and native fungal communities involve both direct antagonism (e.g., antifungal compound secretion) and indirect competition (nutrient depletion, niche exclusion), while synergistic interactions with lactic acid bacteria and other starter cultures may further stabilise the microbial ecosystem. It has been proven that *D. hansenii* strains can significantly inhibit the growth of *Aspergillus* sp. along with aflatoxin production in dry-cured meat products, particularly at lower water activity levels [12]. This yeast also inhibits the growth of *Penicillium* species such as *P. nordicum* and *P. verrucosum*, thereby reducing ochratoxin A (OTA) levels present in infected samples [12,13,14,15].

*D. hansenii’s* biocontrol activity is attributed to multiple mechanisms, including rapid nutrient and space competition, the production of antifungal volatile organic compounds (VOCs), and the secretion of extracellular enzymes that suppress mould development [15,16]. Beyond safety, *D. hansenii* contributes to flavour development through proteolysis and lipolysis, and its halotolerance makes it particularly well suited to the high-salt, low water activity conditions of dry-cured meats [6]. The use of *D. hansenii* as a starter culture in the production of cured meats not only enhances the sensory qualities but also contributes to the overall safety and stability of the product. Its ability to thrive in high salt concentrations and low-pH environments makes it particularly suitable for the fermentation of cured meats [6]. As research continues, the potential applications of *D. hansenii* in the meat industry are likely to expand, offering new opportunities for improving the quality and safety of cured meat products. The use of *D. hansenii* works as part of a hurdle technology approach. Its capability to act as a biocontrol agent within reduced levels of chemical preservatives, while maintaining the quality of the product, is particularly attractive for the industry. Hurdle technology refers to the combined use of multiple, individually mild preservation factors to collectively ensure product safety without compromising quality [16]. Integrating *D. hansenii* inoculation into existing industrial curing workflows offers a natural, cost-effective, and consumer-friendly strategy to complement or partially replace synthetic preservatives such as nitrites.

Previous studies in our group showed that the presence of autochthonous *D. hansenii* strains [6,17] results in improved final security levels of the products even when preservatives like nitrites are reduced. This can be appreciated in the decrease of the presence of potential pathogenic microorganisms like filamentous fungi [17]. These changes have been studied with the aim of protecting consumer health, but deepening studies into the effects of this yeast on the microbiome of the Iberian pork loin have not been performed yet. Given the exponential growth of metabarcoding and amplicon-based studies, one would expect there to be extensive knowledge of the pork loin microbial communities. Despite this and the high popularity of Spanish Iberian pork loin among consumers, the native microbial communities that develop during its curing process and contribute to its unique flavour and texture have not been extensively studied. The use of high-throughput sequencing (HTS) technology could provide insights into the microbial diversity present in Iberian pork loin. HTS can generate thousands of sequence reads, potentially identifying complex microbial consortia, including low-abundance microorganisms. Over the past decade, HTS techniques have been employed to characterise the microbial communities in various cured meat products worldwide, revealing the intricate microbial ecosystems that influence their sensory properties [18,19]. Taking into consideration the study conducted by Kamilari et al. [20], where several DNA extraction protocols were tested to study Cyprus sausages’ bacterial community identification processes, in the present work DNA extraction followed by Ion Torrent sequencing was performed to study the effect of *D. hansenii* LR2 inoculation in the fungal biodiversity of Iberian Pork Loins after the curing process. The main contribution of the present research is to provide a snapshot of the Iberian pork loin fungal communities. The main objective of this study was to evaluate the impact of *D. hansenii* LR2 inoculation on the fungal community structure of Iberian pork loin using ITS1 metabarcoding, with the specific aim of determining whether this yeast can suppress spoilage and mycotoxin-producing fungi under curing conditions. Our results confirm that the presence of *D. hansenii* modifies the fungal population of meat products, significantly reducing the presence of potentially pathogenic genera such as *Aspergillus* or *Penicillium*, among others.

## 2. Materials and Methods

### 2.1. Inoculation and Sample Collection

In this study, two types of batches of Iberian pork loins were used: an inoculated batch (CL) with *D. hansenii* LR2, a strain isolated from Iberian pork loins [21], and a non-inoculated batch (SL) cured in the traditional way. The inoculation was made following the method described by Chacón-Navarrete et al. [17]. In the cited work the studied strain demonstrated to significantly reduce mould populations and inhibit toxigenic fungi through volatile compound production. In it, *D. hansenii* LR2 cells were grown in Yeast Maltose Broth media (YMB) from Millipore (Darmstadt, Germany) composed of glucose (1%), maltose extract (0.3%), peptone (0.5%) and yeast extract (0.3%) with an addition of 3% NaCl. Cultured cells were collected by decanting and subsequently washed by resuspending in distilled water. The cell suspension was maintained at a low temperature until use. Final inoculum suspension had approximately 0.2 g/mL (~10^6^ CFU/mL) of yeast cells. Loin samples were taken after three months of curing, transported in ice-cool packs and stored at −20 °C until processing.

### 2.2. DNA Extraction

Loins morphology leads to three differentiated parts, the top, the middle and the bottom. Top and bottom parts near the closure section of the casing usually develop into drier zones. The bottom section also tends to accumulate more marinade and, when inoculated, potentially more yeast. To account for these heterogeneities, samples from each section were pooled to create.

**Figure 1:**
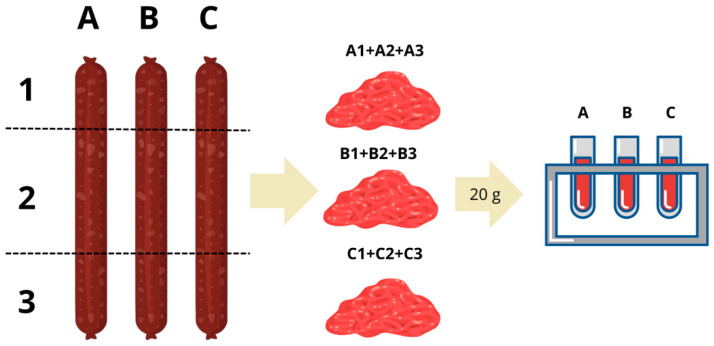
Graphical representation of the sampling strategy applied to Iberian pork loins. Each loin piece (A, B, C; representing the three biological replicates) was divided into three sections: Section 1 (top/closure zone, typically drier), Section 2 (central portion), and Section 3 (bottom zone, which accumulates more marinade and, in inoculated loins, potentially more yeast inoculum). Portions from each section were pooled to generate a single composite homogenate per loin, from which 20 g aliquots were taken and suspended in 180 mL of sterile Buffered Peptone Water (BPW) for microbial analysis. This was performed for CL ans SL samples. CL: inoculated batch. SL: non-inoculated control batch.

Composite homogenates. Pieces of each of those sections were unified in a pool, from which portions of 20 g for each replicate were taken and suspended in 180 mL of sterile Buffered Peptone Water (BPW) (Figure 1). Three replicates were performed per batch. Loin pieces were randomly selected from a commercial production batch of over 200 units. To ensure spatial representativeness within the curing chamber, one loin was taken from each of three distinct shelf positions corresponding to different microclimatic zones: the central area, the lateral zone, and the entrance of the curing chamber. This sampling strategy was designed to account for potential environmental gradients (airflow, humidity, temperature) that may exist within industrial curing facilities, thereby minimising spatial bias in the microbial analysis.

Samples were then homogenised using a Stomacher for 5 min at 300 rpm. Then, 1 mL of the solution was transferred to 2 mL Eppendorf tubes and centrifuged for 1 min at 14,000 rpm. The supernatant was discarded. NucleoSpin Food of Macherey-Nagel for extraction of total DNA was used in combination with an additional bead-beating step during sample homogenisation according to previous information described by Kamilari et al. 2021 [20]. The DNA extraction process was performed according to the kit manufacturer’s instructions. The extracted DNA was stored at −20 °C until processing.

### 2.3. Quantification of Total DNA

The total amount and purity of DNA extracted from the Iberian pork loins were quantified using a Nanodrop 2000C. The DNA purity was assessed by measuring the absorbance ratios A_260_/_280_ nm and A_260_/_230_ nm.

### 2.4. Isolation and Identification of Culturable Fungi

Meat samples were minced and homogenised with buffered peptone using a Stomacher. Homogenised mixtures were transferred to new tubes, and serial dilutions were performed in Sabouraud Chloramphenicol Agar (Condalab, Torrejón de Ardoz, Madrid, Spain). Plates were incubated under aerobic conditions at 26 °C for 5 days until visible colonies developed. Isolated colonies of each microorganism were subjected to a DNA extraction protocol with Chelex resin. Attending to the type of microorganism; moulds or yeasts, different genomic DNA extraction protocols was carried out. For yeasts, a protocol based on Blount et al. [22] was applied. Briefly, a single colony was transferred into a 1.5 mL microcentrifuge tube and resuspended in 100 µL of 5% Chelex solution. Acid-washed glass beads were added up to approximately half of the sample volume, followed by vortexing for 4 min. Samples were then incubated at 100 °C for 2 min, centrifuged for 1 min at maximum speed, and the supernatant containing DNA was transferred to a new tube.

For filamentous fungi, DNA extraction was carried out following Conlon et al. [23]. Approximately 10 mg of mycelium was scraped from the agar surface using a sterile scalpel and transferred to a pre-weighed 1.5 mL tube. Mycelium was resuspended in 200 µL of 5% Chelex solution supplemented with 10 µL of proteinase K (20 mg/mL), vortexed for 15 s, and incubated at 65 °C for 30 min. Samples were then centrifuged at 3300× *g* for 3 min, and 100 µL of the supernatant was recovered for downstream analysis.

Molecular identification of isolates was performed by amplification of the internal transcribed spacer (ITS1) region. PCR reactions were carried out in a final volume of 50 µL using MyTaq^TM^ HS Red Mix (Bioline Meridian, Memphis, TN, USA), following the manufacturer’s recommendations. The reaction mixture contained approximately 200 ng of template DNA and 1 µL of each primer (20 µM). The primers used were *ITS1-30F* (5′-GTCCCTGCCCTTTGTACACA-3′) and *ITS1-217R* (5′-TTTCGCTGCGTTCTTCATCG-3′) [24]. PCR cycling conditions consisted of an initial denaturation at 95 °C for 1 min, followed by 30 cycles of denaturation at 95 °C for 15 s, annealing at 55 °C for 15 s, and extension at 72 °C for 10 s. Finally, PCR products were subjected to Sanger sequencing. Taxonomic assignments were manually curated and validated using MycoBank (https://www.mycobank.org/, accessed on 3 May 2026), and nomenclature was updated according to current taxonomy when necessary.

### 2.5. Microbiome and Statistical Analysis

All samples were tested for amplification by PCR for the DNA Internal Transcribed Spacer region *ITS1*. PCR reactions were carried out using 10 ng/μL of the extracted DNA. Primers used for *ITS1* amplification were: *ITS1-30F*: 5′-GTCCCTGCCCTTTGTACACA-3′ and *ITS1-217R*: 5′- TTTCGCTGCGTTCTTCATCG-3′ [24]. PCR conditions were 95 °C for 5 min; 30 cycles of 95 °C for 1 min, 55 °C for 1 min, and 72 °C for 2 min; and 72 °C for 10 min.

Amplicons were sequenced using the Ion Torrent massive sequencing method at the SCAI (Central Research Support Service) facilities of the University of Córdoba (Spain), generating single-end FASTQ files in Phred33 format. Primer sequences were trimmed using the Python 2.7 script *Split_on_primer.py*, allowing a maximum of 5 mismatches for primer matching. The resulting FASTQ files were analysed using the QIIME 2 pipeline (version 2024.5) with Python 3.10.14 [25]. Sequence quality was initially assessed using the QIIME 2 demux summarise function. The QIIME 2 *ITSxpress* plugin [26] was then used to remove the *ITS1* conserved regions to achieve a higher taxonomic resolution. Subsequently, DNA sequences were denoised using DADA2 without applying additional fixed trimming or truncation thresholds, as quality profiles showed consistently high-quality reads across the full amplicon length and *ITSxpress* had already standardised *ITS1* sequence boundaries. DADA2 performed quality filtering based on its expected error model, including dereplication, phiX filtering, and chimaera removal. Representative sequences were then clustered at 99% identity into operational taxonomic units (OTUs) using VSEARCH. Taxonomical classification was carried out creating a UNITE (version 10.0) [27] classifier for the *ITS1* region. Data analysis and diversity calculations were carried out using the R package *mia* (version 1.15.4).

Taxonomic data was filtered applying prevalence and detection thresholds of 20% and 0.1%, respectively. Alpha diversity indexes of Shannon–Wiener (H’), Pielou (J’) and Gini–Simpson (D’) were calculated at the family level and followed by Welch’s t-tests and Benjamini–Hochberg correction to assess the differences between inoculated and non-inoculated samples. MDS analysis was performed at the family level using the Aitchison distance for the assessment of beta diversity. A PERMANOVA test setting 9999 permutations was carried out to evaluate the differences between the two conditions. Differential abundance analysis at the genus level was performed using the R package *ALDEx2* (version 1.38.0) [28]. To do so, 1000 Monte-Carlo instances were generated using the *aldex.clr* function, followed by centred log-ratio (CLR) transformation to account for the compositional nature of sequencing data. Effect sizes were estimated using *aldex.effect*, and significant differential abundance was determined using a size effect threshold of |effect| > 1. Clustering at the species level was carried out using the R package *sechm* (version 1.14.0). To do so, relative abundances of the number of identifications were calculated, and data was row-scaled and hierarchically clustered. Graphics were generated using the ggplot2 R package (version 4.0.2) and Excel.

## 3. Results

### 3.1. Operational Taxonomic Units (OTUs) and Culturable Isolates Comparison

The OTU clustering revealed a total of 55,157 reads assigned to 41 OTUs (Table S1). The OTU 40 was excluded for further analysis, as its *Incertae Sedis* taxonomic status introduced bias in the statistical analysis at the genus and species levels. OTU 41 was also removed since it was only identified at the kingdom level. Following data filtering, the comparison between culturable and sequencing-based identification revealed clear differences between both approaches (Table 1).

When comparing culture-based identification with high-throughput sequencing, clear divergences emerge at both taxonomic resolutions. At the genus level, these patterns persist but become less extreme: three genera were exclusively cultured, whereas 21 appeared solely in sequencing data. The shared core of four genera highlights a set of taxa both amenable to in vitro growth and readily captured by sequence-based assays. At the species level, only three taxa (Table 1) were uniquely isolated by traditional culture. In contrast, sequencing alone detected a much richer diversity with a higher number of species. Only four genera were consistently identified by both approaches—*Candida*, *Pichia*, *Debaryomyces*, and *Aspergillus*—underscoring a modest intersection in detection (Table 1).

**Figure 2:**
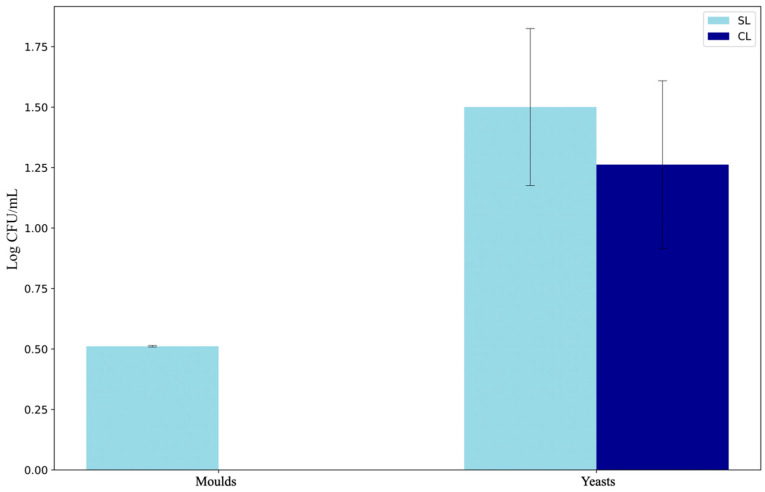
Total microbial counts (expressed as Log CFU/mL) of moulds and yeasts in Iberian pork loin samples without *D. hansenii* inoculum (SL, non-inoculated control) and with *D. hansenii* inoculum (CL, inoculated), grown on Sabouraud Chloramphenicol Agar. Bars represent mean values of three biological replicates. Mould counts in inoculated samples (CL) were below the detection limit (0 CFU/mL) and are therefore not shown. Error bars indicate standard deviation.

In addition, when contrasting the amplicon sequencing results with microbial counts in Sabouraud Chloramphenicol Agar, the conclusions align, as we observe a significant decrease in mould populations. This decline is particularly striking as moulds not only diminish but entirely disappear (Figure 2).

### 3.2. Biodiversity Index Evaluation

**Figure 3:**
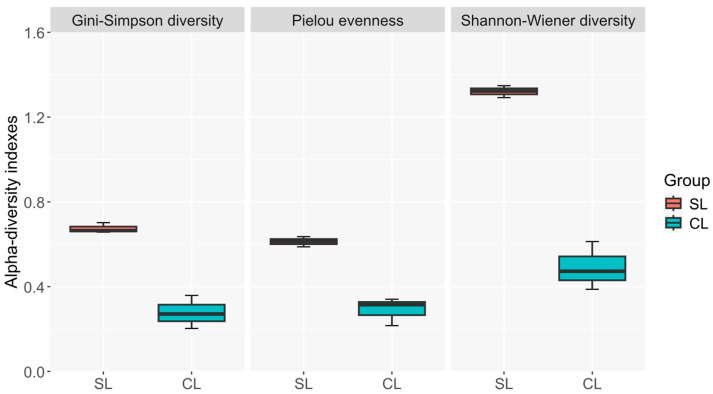
Box-plot graphs of alpha diversity indices: Shannon–Wiener (H’), Pielou (J’), and Gini–Simpson (D’) calculated at the family level for non-inoculated (SL) and *D. hansenii*-inoculated (CL) Iberian pork loin samples (*n* = 3 per condition). All three indices show consistently lower values in inoculated samples, indicating reduced fungal diversity and evenness following yeast addition. Statistically significant differences between groups were confirmed by Welch’s *t*-test: Shannon (*p* = 0.0042), Pielou (*p* = 0.0075), Gini–Simpson (*p* = 0.0081).

To study the alpha diversity of the samples the indexes of Shannon–Wiener, Pielou and Gini–Simpson were calculated at the family level (Figure 3).

In a first approach, indexes had very similar tendencies after filtering by prevalence (20/100) and by detection (0.1/100), as shown in Table S2. Welch’s *t*-tests, however, revealed statistically significant differences between CL and SL conditions for all three indices: Shannon (*p* = 0.0042), Pielou (*p* = 0.0075), and Gini–Simpson (*p* = 0.0081). These results confirm that samples inoculated with *D. hansenii* (CL samples) exhibited significantly lower diversity and evenness compared to the non-inoculated controls (SL samples), as depicted in Figure 3.

**Figure 4:**
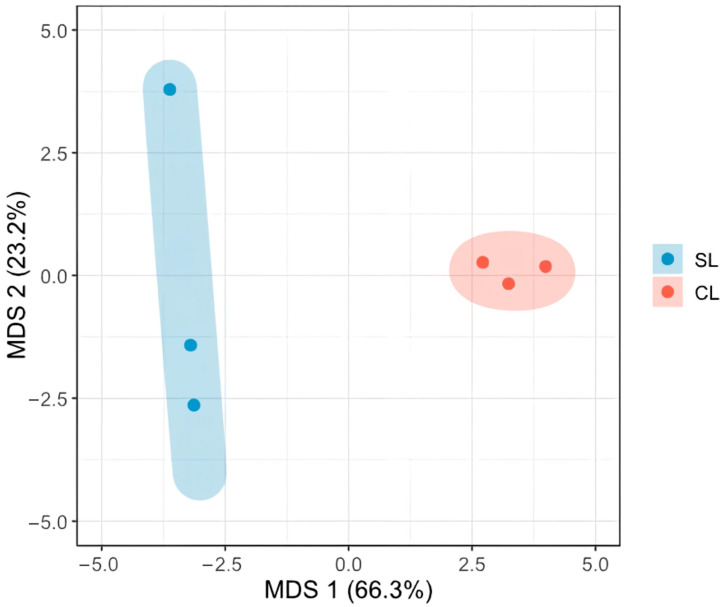
Multidimensional scaling (MDS) representation based on Aitchison distance, illustrating the differentiation in fungal community structure between samples inoculated with *D. hansenii* (CL) and non-inoculated control samples (SL).

To further analyse the global structure of the fungal community after inoculation with *D. hansenii*, a beta diversity study was performed using non-metric multidimensional scaling analysis (MDS) based on the Aitchison distance (Figure 4). This method is widely used to analyse compositional data from amplicon sequencing, since it appropriately handles the relative nature of microbiological data [29]. Although the PERMANOVA test did not reveal significant differences between conditions, the graphical representation suggests a marked difference between samples inoculated with *D. hansenii* (CL) and non-inoculated samples (SL), indicating that the introduction of the starter culture altered the global composition of the microbial community.

### 3.3. Genus Variability: D. hansenii Inoculation Influence

**Figure 5:**
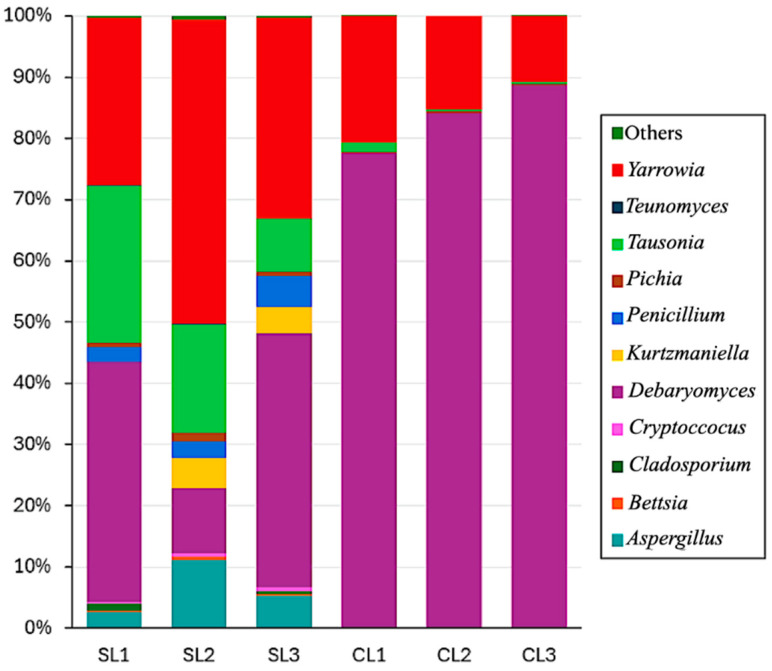
Stacked bar chart of relative genus-level abundance of fungi in Iberian pork loin samples with (CL, right) and without (SL, left) *D. hansenii* inoculum. Each bar represents one biological replicate. *Debaryomyces* and *Yarrowia* are the dominant genera across both conditions; inoculation substantially increases *Debaryomyces* relative abundance while reducing *Yarrowia*, *Aspergillus*, *Penicillium*, *Pichia*, and *Teunomyces*. Genera representing <1% relative abundance across all samples are grouped as “Other.”.

A more detailed examination of the microbial diversity modifications after the addition of *D. hansenii* during the curing process of Iberian Pork Loin revealed interesting outcomes (Figure 5).

A preliminary inspection of the data representation highlights that *Debaryomyces and Yarrowia* are the main genera present in inoculated and non-inoculated samples. As expected, after the inoculation, *Debaryomyces* became the main represented genus in the samples. However, *Yarrowia* remained a relevant component of the fungal community, although its relative abundance decreased from approximately 37% in control samples to 15% in inoculated samples (Figure 5). At the species level, this genus was dominated by *Y. alimentaria*, which accounted for approximately 95% of the *Yarrowia* population in both control and inoculated samples. This shift was accompanied by a marked decrease in *Tausonia* from 17% to 0.7%, a moderate reduction in *Pichia* from 0.90% to 0.25%, and almost complete or complete reduction of *Penicillium*, *Aspergillus*, and *Teunomyces* (Table S1).

### 3.4. Species Variability: D. hansenii Inoculation Influence

**Figure 6:**
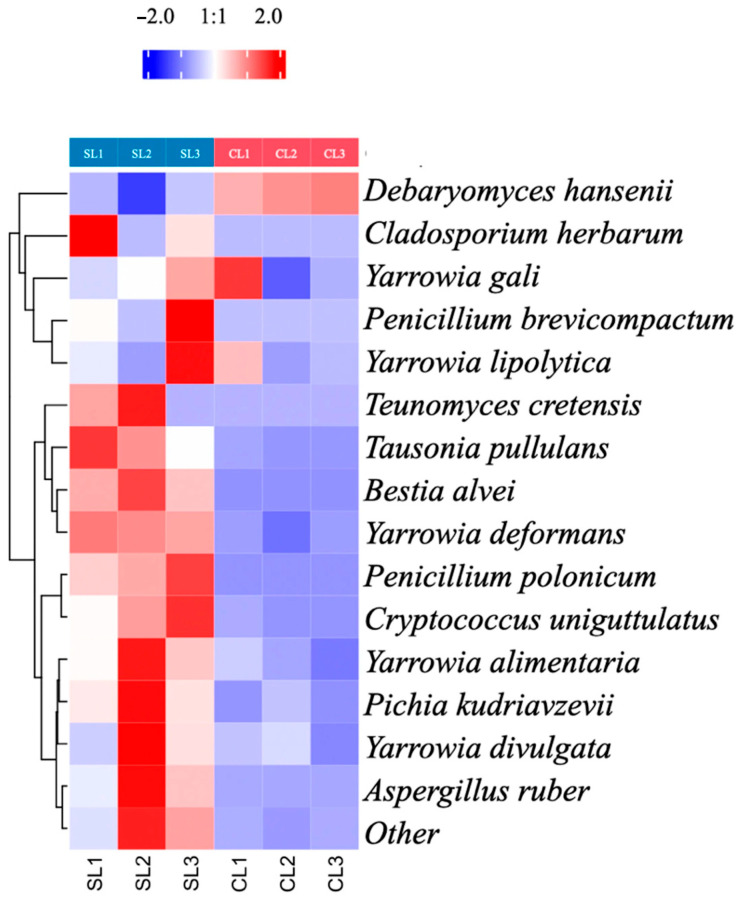
Heatmap of species-level variation in relative fungal abundance in Iberian pork loins with (CL) and without (SL) *D. hansenii* inoculum (*n* = 3 per condition). Colour scale ranges from −2 (blue; lower relative abundance) to +2 (red; higher relative abundance), based on scaled relative read counts. Hierarchical clustering dendrograms (rows, species; columns, samples) were generated using the *sechm* R package. Clear separation between CL and SL samples confirms a pronounced reorganisation of the fungal community following inoculation. Species associated with spoilage or mycotoxin production (e.g., *Aspergillus* and *Penicillium* spp.) appear at lower abundance (blue) in inoculated samples.

Taking a step further into the analysis, the species-specific variability after *D. hansenii* inoculation was studied. To do so, we present a heatmap in Figure 6 where the range was adjusted to −2 and 2, and a hierarchical analysis was performed using the sechm R package 4.6.0.

A clear visual separation can be observed between the inoculated (CL) and control groups (SL), as shown by the hierarchical clustering dendrograms flanking the heatmap. These dendrograms confirmed that the inoculation with *D. hansenii* significantly altered the fungal ecology of the system as suggested in previous analysis. More specifically, the abundance of several fungal species associated with spoilage or potential mycotoxin production was noticeably reduced in the treated samples (blue-coloured cells), aligning with the genus-level study. In contrast, these same taxa appeared in greater abundance (red cells) in the non-inoculated samples, implying that their proliferation was suppressed in the presence of *D. hansenii*. Conversely, *D. hansenii* itself was detected in higher presence in the inoculated samples, confirming successful colonisation. The observed reduction in key mycotoxin-producing species (*Aspergillus* and *Penicillium*) may contribute to mitigating fungal growth and potentially reducing mycotoxin-associated risks, pending direct toxin quantification in future research.

## 4. Discussion

Taking into consideration the aim of this study of evaluating whether the inoculation of *D. hansenii* LR2 could alter the native fungal biodiversity of Iberian pork loins, while preventing the proliferation of unwanted fungi, the obtained results clearly demonstrate that this yeast exerts a remarkable influence on the cured meat mycobiota. The amplicon-based sequencing approach applied, combining Ion Torrent sequencing with classical culture methods, provided a detailed snapshot of the fungal communities associated with Iberian pork loins after the curing process. This integrated strategy allowed the detection of both culturable and unculturable taxa, revealing substantial shifts in community composition followed by yeast inoculation. In particular, the presence of *D. hansenii* significantly reduced the abundance of potentially pathogenic and spoilage genera such as *Aspergillus* and *Penicillium*, confirming its strong competitive and biocontrol action. To our knowledge, this is the first study to characterise the fungal community of Iberian pork loin under *D. hansenii* inoculation using an *ITS1* metabarcoding approach. Consistent with the culture-based evidence reported in our previous work [17], the metabarcoding data confirm that *D. hansenii* LR2 reproducibly suppresses mould populations at the whole-community level, extending prior findings from traditional microbiological assays to the amplicon sequencing context, although the observed microbial shifts should be interpreted as strongly associated with the presence of *D. hansenii* rather than as definitive proof of causality. Nevertheless, these findings are consistent with a substantial body of literature demonstrating the biocontrol activity of *D. hansenii* and its role in shaping fungal community structure [12,14,15,16].

The apparent discrepancies between culture-dependent and sequencing results reflect the importance of what has been described by other authors: combining culture and sequencing is essential to fully uncover microbial diversity [30,31,32]. Culture methods tend to favour fast-growing, aerotolerant fungal species capable of thriving on artificial media, whereas sequencing uncovers a broader spectrum of taxa, including slow-growing or uncultivable ones. Results showed a substantial increase in unique genera and species detected exclusively by sequencing methods, underscoring its superior sensitivity for community profiling. The small set of culture-only taxa detected suggests niche organisms that may be outcompeted in mixed environmental samples but flourish in isolation. The dual approach, therefore, maximises taxonomic coverage and leverages the strengths of each method to construct a more complete picture of the fungal community structure. It should be noted that Table 1 is intended as a qualitative overview of the results. The purpose of this table is to illustrate the extent of taxonomic coverage achieved by each approach, not to formally quantify overlap significance. As previously argued in the literature, direct statistical comparison of culture-based and sequencing-based detection is methodologically limited by the fundamentally different nature of the two techniques, which capture distinct and not fully overlapping fractions of the mycobiome [31,32,33].

In our study, Welch’s *t*-tests revealed statistically significant differences in all alpha diversity indices between inoculated and non-inoculated samples, confirming that *D. hansenii* inoculation led to a significant reduction in both richness and evenness of the fungal community. Such a decrease in alpha diversity is a common outcome in fermented food systems where a specific microorganism is introduced to standardise fermentation, often resulting in a more predictable and less diverse microbial ecosystem [33,34,35]. The visual separation observed in the MDS plot further supports the hypothesis of a compositional shift in the community structure. Despite the evident separation between clusters, the dispersion observed within treatments (particularly in the control samples) likely reflects the intrinsic variability of artisanal curing processes and the limited number of biological replicates available. This variability is therefore most plausibly attributed to biological heterogeneity inherent to the production system, although a contribution of technical variability associated with sample processing and sequencing cannot be completely excluded. While three biological replicates per condition represent a limitation, this study was designed as a first exploratory approach to characterise the fungal community dynamics of Iberian pork loin under *D. hansenii* inoculation, a question that had not previously been addressed using metabarcoding. The results provide a solid foundation for future studies with expanded sample sizes and replicated batches. Interestingly, inoculation with *D. hansenii* not only reduced the abundance of unwanted fungal genera as described below but also resulted in a visually more homogeneous clustering pattern among samples in the MDS analysis. This aligns with previous studies describing how inoculated yeasts dominate and stabilise microbial communities in fermented products, displacing potentially harmful or unwanted species [12,15,36].

The yeast inoculum treatment led to a marked dominance of *D. hansenii* among the yeast community, accompanied by a reduction in genera such as *Yarrowia*, *Pichia*, *Tausonia*, and *Teunomyces*. Among these yeasts, *Teunomyces* has not been commonly described in this context, *Tausonia* is less frequently reported in dry-cured meat products [32,33], whereas *Yarrowia* and *Pichia* are commonly found in these systems [37,38,39], where they may play both beneficial and detrimental roles depending on their abundance and metabolic activity. *Yarrowia alimentaria* and *Y. lipolytica*, yeasts, commonly found in dairy, meat, and fermented foods, are valued for their proteolytic and lipolytic activities that contribute to flavour development [38,40,41,42]. However, their overgrowth can cause off flavours. Their decline in the presence of *D. hansenii* likely reflects competition for ecological niches, without detrimental impact on product quality [17]. As *Yarrowia* abundance decreased in inoculated samples, but was not entirely suppressed, this suggests a selective reduction rather than complete exclusion. Furthermore, different *Yarrowia* species may be represented in each condition, as the genus-level analysis does not distinguish between beneficial *Yarrowia* species and other species with different functional roles. Species-level resolution from the heatmap (Figure 6) partially addresses this. Similarly, *Pichia* species, while sometimes contributing positively to fermentation processes, may also be associated with film formation and spoilage [43]. The observed reduction in these genera suggests that *D. hansenii* exerts a selective pressure on the yeast community, modulating the abundance of both beneficial and undesirable taxa rather than indiscriminately eliminating them.

Regarding the filamentous fungi population, the disappearance or drastic reduction of *Aspergillus* and *Penicillium* after inoculation is particularly relevant from a food safety perspective, since these genera are responsible for spoilage and mycotoxin production [44,45]. These findings are consistent with previous works reporting the antimycotic potential of *D. hansenii* via competition for nutrients and space, secretion of antifungal compounds, and production of volatile organic compounds that suppress mould development [12,14,15,16]. The decrease observed in *Bettsia* abundance is noteworthy. *Bettsia* species are filamentous fungi typically associated with bee hives [46,47,48]. Their occurrence in meat samples may relate to environmental exposure in the curing facilities. Interestingly, *D. hansenii* has been reported in similar niches [38], suggesting a broader ecological versatility and potential for novel applications as an antimycotic agent beyond the food industry.

Altogether, our findings clearly demonstrate that the inoculation of *D. hansenii* substantially modifies the fungal community structure of Iberian pork loins, resulting in a pronounced reduction of potentially spoilage and mycotoxin-producing genera such as *Aspergillus* and *Penicillium*. These findings offer a representative insight into artisanal and controlled industrial systems, identifying *D. hansenii* as a significant factor in the process. This work establishes a solid foundation for future research to confirm these trends across expanded environmental and production scales. The yeast treatment also promoted a more homogeneous microbial profile among samples, suggesting a stabilising effect on the curing ecosystem and contributing to the standardisation of the final product. Although the present study did not directly investigate the molecular or biochemical mechanisms underlying this selective pressure, the observed trends are in line with previously reported antagonistic activities of *D. hansenii*, which are thought to involve rapid growth, efficient nutrient utilisation, and the production of antifungal compounds [12,15,17,36] as well as the ability to reduce mycotoxin accumulation, such as ochratoxin A, when applied either alone or in combination with other microorganisms or plant-derived extract [49,50]. Recent work has specifically evaluated the role of nutrient competition as a key driver of *D. hansenii* biocontrol activity against spoilage moulds in the meat industry, providing experimental support for this mechanistic hypothesis [51], as well as highlighting the importance of using food-mimicking media to accurately assess the biocontrol potential of different strains [52]. In this context, our results reinforce the potential of *D. hansenii* as an effective biocontrol agent in dry-cured meat products, combining the advantages of improved microbial safety and reduced variability between batches with the added benefit of a natural and sustainable preservative strategy.

## 5. Conclusions

This study highlights the substantial potential of the yeast *D. hansenii* as an effective starter culture for biocontrol in the production of Iberian cured pork loin. Our results clearly demonstrate that inoculation with *D. hansenii* significantly reshapes the fungal community structure, reducing the prevalence of fungi commonly associated with spoilage and mycotoxin production, particularly genera such as *Aspergillus* and *Penicillium*. The dominance of *D. hansenii* within the microbial community reduces the abundance of spoilage and potentially toxigenic genera, an indirect indicator of improved microbial safety. However, direct mycotoxin quantification in future studies would further validate this claim. Amplicon sequencing provided detailed insights into the microbial interactions occurring during the curing process, highlighting the competitive dominance of *D. hansenii*. These findings align with previous research, reinforcing the role of *D. hansenii* as a natural preservative and a promising alternative to chemical preservatives. Furthermore, our results emphasise the need to deepen our understanding of microbial community dynamics to optimise fermented food production, safety, and quality through sustainable biocontrol strategies. Building on these results, subsequent studies will allow for the validation of these patterns at a broader industrial scale, ensuring their consistency across different production environments.

## Figures and Tables

**Table 1:** List of identified fungal species.

Only Culture	Sequencing and Culture Identification	Only Sequencing	
*Debaryomyces castelli*	*Aspergillus flavus*	*Aspergillus ruber*	*Sampaiozyma* sp.
*Rhodotorula mucilaginosa*	*Candida albicans*	*Bettsia alvei*	*Schizosaccharomyces pombe*
	*Candida zeylanoides*	*Cladosporium herbarum*	*Sporobolomyces reniformis*
	*Debaryomyces hansenii*	*Colletotrichum coccodes*	*Tausonia pullulans*
	*Pichia kudriavzevii*	*Cryptococcus uniguttulatus*	*Teunomyces cretensis*
		*Eremascus albus*	*Verticillium dahlia*
		*Mrakia frigida*	*Vishniacozyma dimennae*
		*Neocamarosporium endophyticum*	*Vishniacozyma heimaeyensis*
		*Penicillium bialowiezense*	*Vishniacozyma victoriae*
		*Penicillium brevicompactum*	*Yarrowia alimentaria*
		*Penicillium polonicum*	*Yarrowia bubula*
		*Pichia kluyveri*	*Yarrowia deformans*
		*Preussia polymorpha*	*Yarrowia divulgata*
		*Pseudogymnoascus appendiculatus*	*Yarrowia galli*
		*Rhizophydiomycetes* sp.	*Yarrowia lipolytica*

## Data Availability

The original contributions presented in this study are included in the article/Supplementary Material. Further inquiries can be directed to the corresponding author.

## Supplementary Materials

The following supporting information can be downloaded at: https://www.mdpi.com/article/10.3390/microorganisms14051113/s1, Table S1: OTUs identified through metagenomic analysis. Table S2: Alpha diversity indexes values before and after filtration. SL stands for non-inoculated samples and CL for inoculated ones. Number corresponds to the replica in each case.
