# Supplementary material for: Debaryomyces hansenii Reshapes the Fungal Community of Iberian Cured Pork Loin: An ITS1 Metabarcoding Approach
Source: Microorganisms. 2026 May 14;14(5):1113. doi: 10.3390/microorganisms14051113 (PMC13210324; doi:10.3390/microorganisms14051113)
Supplement: Supplementary file 1 [file microorganisms-14-01113-s001.zip › microorganisms-4294240-supplementary Table S1-S2.pdf]

Table S1. OTUs identified through metagenomic analysis.

OTU	Taxonomy							No. of counts (SL)			No. of counts (CL)		
	Kingdom	Phylum	Class	Order	Family	Genus	Species	1	2	3	1	2	3
OTU 1	Fungi	Ascomycota	Dothideomycetes	Cladosporiales	Cladosporiaceae	*Cladosporium*	*Cladosporium herbarum*	57	0	15	0	0	0
OTU 2	Fungi	Ascomycota	Dothideomycetes	Pleosporales	Neocamarosporiaceae	*Neocamarosporium*	*Neocamarosporium endophyticum*	0	4	0	0	0	0
OTU 3	Fungi	Ascomycota	Dothideomycetes	Pleosporales	Sporormiaceae	*Preussia*	*Preussia polymorpha*	0	0	0	2	0	0
OTU 4	Fungi	Ascomycota	Eurotiomycetes	Ascosphaerales	Ascosphaeraceae	*Bettsia*	*Bettsia alvei*	22	48	16	0	0	0
OTU 5	Fungi	Ascomycota	Eurotiomycetes	Coryneliales	Eremascaceae	*Eremascus*	*Eremascus albus*	0	9	0	0	0	0
OTU 6	Fungi	Ascomycota	Eurotiomycetes	Eurotiales	Aspergillaceae	*Aspergillus*	*Aspergillus flavus*	0	3	0	0	0	0
OTU 7	Fungi	Ascomycota	Eurotiomycetes	Eurotiales	Aspergillaceae	*Aspergillus*	*Aspergillus ruber*	139	934	252	4	0	0
OTU 8	Fungi	Ascomycota	Eurotiomycetes	Eurotiales	Aspergillaceae	*Aspergillus*		0	0	0	0	0	5
OTU 9	Fungi	Ascomycota	Eurotiomycetes	Eurotiales	Aspergillaceae	*Penicillium*	*Penicillium bialowiezense*	0	22	0	0	0	0
OTU 10	Fungi	Ascomycota	Eurotiomycetes	Eurotiales	Aspergillaceae	*Penicillium*	*Penicillium brevicompactum*	18	0	72	0	0	0
OTU 11	Fungi	Ascomycota	Eurotiomycetes	Eurotiales	Aspergillaceae	*Penicillium*	*Penicillium polonicum*	111	201	172	0	0	0
OTU 12	Fungi	Ascomycota	Leotiomycetes	Thelebolales	Pseudeurotiaceae	*Pseudogymnoascus*	*Pseudogymnoascus appendiculatus*	6	0	0	0	0	0
OTU 13	Fungi	Ascomycota	Saccharomycetes	Saccharomycetales	Debaryomycetaceae	*Debaryomyces*	*Debaryomyces hansenii*	2140	898	1965	5880	4622	6310
OTU 14	Fungi	Ascomycota	Saccharomycetes	Saccharomycetales	Debaryomycetaceae	*Kurtzmaniella*		0	417	205	0	0	0
OTU 15	Fungi	Ascomycota	Saccharomycetes	Saccharomycetales	Pichiaceae	*Pichia*	*Pichia kluyveri*	0	9	0	0	0	0
OTU 16	Fungi	Ascomycota	Saccharomycetes	Saccharomycetales	Pichiaceae	*Pichia*	*Pichia kudriavzevii*	37	114	32	15	20	13
OTU 17	Fungi	Ascomycota	Saccharomycetes	Saccharomycetales	Saccharomycetales fam Incertae sedis	*Candida*	*Candida albicans*	0	14	0	0	0	0
OTU 18	Fungi	Ascomycota	Saccharomycetes	Saccharomycetales	Saccharomycetales fam Incertae sedis	*Teunomyces*	*Teunomyces cretensis*	6	15	0	0	0	0
OTU 19	Fungi	Ascomycota	Saccharomycetes	Saccharomycetales	Saccharomycetales fam Incertae sedis	*Yarrowia*	*Yarrowia alimentaria*	1418	4026	1455	1494	831	737
OTU 20	Fungi	Ascomycota	Saccharomycetes	Saccharomycetales	Saccharomycetales fam Incertae sedis	*Yarrowia*	*Yarrowia bubula*	0	6	19	0	0	0
OTU 21	Fungi	Ascomycota	Saccharomycetes	Saccharomycetales	Saccharomycetales fam Incertae sedis	*Yarrowia*	*Yarrowia deformans*	59	81	42	13	0	12

OTU 22	Fungi	Ascomycota	Saccharomycetes	Saccharomycetales	Saccharomycetales fam Incertae sedis	*Yarrowia*	*Yarrowia divulgata*	6	45	11	7	7	0
OTU 23	Fungi	Ascomycota	Saccharomycetes	Saccharomycetales	Saccharomycetales fam Incertae sedis	*Yarrowia*	*Yarrowia galli*	10	20	18	42	0	9
OTU 24	Fungi	Ascomycota	Saccharomycetes	Saccharomycetales	Saccharomycetales fam Incertae sedis	*Yarrowia*	*Yarrowia lipolytica*	4	0	14	12	0	2
OTU 25	Fungi	Ascomycota	Saccharomycetes	Saccharomycetales	Saccharomycetales fam Incertae sedis			282	431	83	22	7	0
OTU 26	Fungi	Ascomycota	Schizosaccharomycetes	Schizosaccharomycetales	Schizosaccharomycetaceae	*Schizosaccharomyces*	*Schizosaccharomyces pombe*	0	0	0	3	0	0
OTU 27	Fungi	Ascomycota	Sordariomycetes	Glomerellales	Glomerellaceae	*Colletotrichum*	*Colletotrichum coccodes*	5	5	7	0	0	0
OTU 28	Fungi	Ascomycota	Sordariomycetes	Glomerellales	Plectosphaerellaceae	*Verticillium*	*Verticillium dahliae*	0	3	0	0	0	0
OTU 29	Fungi	Ascomycota	Sordariomycetes	Hypocreales	Nectriaceae	*Fusarium*		0	7	0	0	0	0
OTU 30	Fungi	Ascomycota						0	0	0	0	0	2
OTU 31	Fungi	Basidiomycota	Microbotryomycetes	Microbotryomycetes ord Incertae sedis	Microbotryomycetes fam Incertae sedis	*Sampaiozyma*	*Sampaiozyma sp*	0	5	0	0	0	0
OTU 32	Fungi	Basidiomycota	Microbotryomycetes	Sporidiobolales	Sporidiobolaceae	*Sporobolomyces*	*Sporobolomyces reniformis*	0	0	0	0	0	2
OTU 33	Fungi	Basidiomycota	Tremellomycetes	Cystofilobasidiales	Mrakiaceae	*Mrakia*	*Mrakia frigida*	0	6	0	0	0	0
OTU 34	Fungi	Basidiomycota	Tremellomycetes	Cystofilobasidiales	Mrakiaceae	*Tausonia*	*Tausonia pullulans*	1399	1478	411	111	17	27
OTU 35	Fungi	Basidiomycota	Tremellomycetes	Tremellales	Bulleribasidiaceae	*Vishniacozyma*	*Vishniacozyma dimennae*	2	0	0	0	0	0
OTU 36	Fungi	Basidiomycota	Tremellomycetes	Tremellales	Bulleribasidiaceae	*Vishniacozyma*	*Vishniacozyma heimaeyensis*	0	0	5	0	0	0
OTU 37	Fungi	Basidiomycota	Tremellomycetes	Tremellales	Bulleribasidiaceae	*Vishniacozyma*	*Vishniacozyma victoriae*	0	0	0	0	0	2
OTU 38	Fungi	Basidiomycota	Tremellomycetes	Tremellales	Cryptococcaceae	*Cryptococcus*	*Cryptococcus uniguttulatus*	16	44	37	4	0	0
OTU 39	Fungi	Chytridiomycota	Rhizophydiomycetes	Rhizophydiomycetes ord Incertae sedis	Rhizophydiomycetes fam Incertae sedis	*Rhizophydiomycetes gen Incertae sedis*	*Rhizophydiomycetes sp*	3	9	6	11	0	14
OTU 40	Fungi	Fungi phy Incertae sedis	Fungi cls Incertae sedis	Fungi ord Incertae sedis	Fungi fam Incertae sedis	*Fungi gen Incertae sedis*	*Fungi sp*	3014	7466	3544	331	90	194
OTU 41	Fungi							43	272	162	136	106	109

Table S2. Alpha diversity indexes values before and after filtration. SL stands for non-inoculated samples and CL for inoculated ones. Number corresponds to the replica in each case.

**Sample**	**Alpha diversity indexes**					
	**Shanon**		**Pielou**		**Gini Simpson**	
	**Original**	**Filtered**	**Original**	**Filtered**	**Original**	**Filtered**
SL1	1.35	1.35	0.56	0.61	0.70	0.70
SL2	1.33	1.32	0.52	0.64	0.66	0.66
SL3	1.29	1.29	0.56	0.59	0.66	0.66
CL1	0.61	0.61	0.30	0.32	0.36	0.36
CL2	0.47	0.47	0.34	0.34	0.27	0.27
CL3	0.39	0.39	0.20	0.22	0.20	0.20
